# Using Digital Art and Attachment Priming in a Web-Based Serious Game to Reduce Pain and Social Disconnection in Individuals With Chronic Pain and Loneliness: Randomized Controlled Trial

**DOI:** 10.2196/52294

**Published:** 2024-11-27

**Authors:** Jorge Peña, Ian Koebner, William Weisman

**Affiliations:** 1 Department of Communication, University of California, Davis, 367 Kerr Hall,, Davis, CA, 95616, United States, 1 9167540977; 2 Cultural Agents Initiative, Harvard University, Cambridge, MA, United States

**Keywords:** pain, social disconnection, loneliness, randomized controlled trial, art, museums, virtual reality, serious games, virtual art, chronic pain and loneliness, attachment, priming, mediation, intervention, cyberpsychology, mental health

## Abstract

**Background:**

Arts engagement using virtual reality and serious games represent promising nonpharmacological self-management treatment approaches to chronic pain. This study is the first randomized controlled trial to explore the impact of a web-based serious game that simulated a visit to an art museum on pain and social disconnection among individuals living with chronic pain and loneliness.

**Objective:**

This study aimed to test the joint and separate effects of exposure to digital art and attachment figure priming on pain and social disconnection among individuals living with chronic pain and loneliness.

**Methods:**

This randomized controlled trial used a 2 (digital artwork present and absent) × 2 (secure attachment and avoidant attachment prime) repeated measures factorial web-based experimental design with a hanging control condition. Mediation and moderation analyses examined how feelings about the social world triggered by the artwork and frequency of museum visits impacted the effects of the interventions on pain and social disconnection.

**Results:**

The results are based on 308 participants. Mean age of the participants was 42.78 (SD 13.11; range 18-76) years, and 60.2% (n=186) were women. Posttest pain was lower than pretest pain for the artwork present (*P*=.001) and absent (*P*=.001) conditions. Similarly, posttest pain was lower than pretest pain for the secure (*P*=.001) and avoidant (*P*=.001) attachment priming conditions. Relative to the control group, artwork present (*P*=.001) and absent (*P*=.01) conditions had decreased posttest pain. The secure (*P*=.001) and avoidant (*P*=.001) attachment priming conditions also had lower posttest pain scores relative to the control group. Moreover, social disconnection decreased from pre- to posttest for both the artwork present (*P*=.04) and the secure attachment priming (*P*=.002) conditions. Relative to the control group, posttest social disconnection was lower for the artwork present (*P*=.02) and secure attachment priming condition (*P*=.03). The artwork-secure attachment (*P*=.001) and artwork-avoidant attachment (*P*=.006) conditions had lower posttest pain scores compared with the control group. Social disconnection decreased from pre- to posttest for the artwork-secure attachment (*P*=.01) and no artwork-secure attachment (*P=*.05) conditions. Posttest social disconnection was lower for the artwork-secure attachment condition compared with the control group (*P*=.04). Positive feelings about the social world triggered by artwork exposure and frequency of museum visits in the last year played a mediating and moderating role in these effects. Positive feelings about the social world were associated with decreased pain (*B*=*−*.53) and social disconnection (*B*=−.25), and these effects operated on individuals exposed to digital artwork at low, medium, and high frequency of physical museum visits.

**Conclusions:**

Relative to a control group, visiting a web-based art museum reliably decreased pain and social disconnection among individuals living with chronic pain and loneliness. Engaging with digital artwork that triggers positive feelings about the social world may mitigate the burden of chronic pain.

## Introduction

### Background

Chronic pain is a leading cause of disability globally [1]. In the United States, the National Academy of Sciences estimates that more than 100 million American people live with chronic pain, at an annual cost between US $560 and US $635 billion in direct medical expenses and lost productivity [2]. The International Association for the Study of Pain classifies chronic pain as pain that persists for more than 3 months, that is associated with significant emotional distress or functional disability, and that is not better accounted for by another condition [3].

Despite widespread acknowledgment of chronic pain’s biopsychosocial nature, its dynamic interaction between the social environment and the individual in whom pain is experienced [4] is inadequately addressed within the biomedical model of clinical medicine [5]. Chronic pain often reduces social role functioning and increases the risk of social disconnection, which can negatively impact both pain interference and pain intensity over time [6,7]. The cultural and entertainment sector in general and museums in particular may be valuable public health partners in addressing the social burden of chronic pain [8]. In particular, the arts can play a key role in promoting health and managing illness across the life span [9], including evidence that cultural engagement both reduces the risk of developing [10] and mitigates the social disconnection associated with chronic pain [11,12]. However, chronic pain may impede individuals from physically visiting museums and decrease cultural engagement in general. In this context, web-based museum visits and serious games have additional features favorable to public health programming: they are often free, available 24 hours a day, unrestricted by geography, and more accessible than in-person museum visits [8].

### Technological and Psychological Factors

Virtual reality (VR) is a computer-generated environment designed to modify an individual’s sensorial inputs and experience [13]. Additionally, serious games are ludic experiences designed to go beyond entertainment and intend to increase health, learning, and social change [14]. These technologies have been deployed as treatment approaches for both acute [15-17] and chronic [18-21] pain across the lifecycle [15,22] with promising albeit early results. VR and serious games thus represent promising nonpharmacological self-management treatment approaches for chronic pain [18,20].

Two psychological mechanisms articulate why engagement with the arts enabled by a serious game may alleviate chronic pain and its associated social disconnection. According to the distraction hypothesis, exposure to an engaging stimulus can draw an individual’s attention away from pain [23]. Therefore, exposure to artworks within a web-based museum may decrease pain by reducing the mental resources available to experience it [24-27]. For instance, children who play commercial video games report decreased pain intensity and resting pain and use less pain relief medication relative to the day before playing video games [28]. In addition, mood management theory predicts that individuals use entertainment media and cultural experiences to increase positive mood and diminish negative mood [29]. For instance, digital art exposure may alleviate pain and decrease social disconnection by inducing positive affect [24,28,30] instead of by simply operating as a visual distractor.

Consider that 1-shot exposure to digital art (ie, a Monet painting) decreases loneliness and anxiety primarily by improving positive social affect relative to no art exposure [31]. However, these studies raise placebo and Hawthorne effect concerns because both digital art and no-art exposure had positive effects on well-being [31,32]. Placebo effects refer to improvement resulting from the administration of an inert treatment with no specific therapeutic properties [33]. The Hawthorne effect refers to a landmark study that found that dim or bright light intensity manipulations invariably resulted in increased productivity, suggesting that simply observing and paying attention to intervention participants may result in improved outcomes [33]. Considering this, there is a need to further examine the effect of digital art exposure on chronic pain and social disconnection.

### Individual and Situational Factors

Further, individual differences may influence the exposure effects of VR and serious games [34]*.* Aesthetic responsiveness or trait differences in the capacity to savor art increases liking and meaning triggered by exposure to a digital Monet painting, which then boosts positive mood along with decreasing state anxiety and negative mood [32]. Considering that repeated engagement with museums, galleries, and concerts is linked to increased well-being [35], we examine how individual differences in the frequency of museum visits statistically moderate the direction and strength of the effect of web-based artwork exposure.

In addition, recent interventions have examined the effects of situational factors, such as the pace and mindset of individuals engaging with digital art. More specifically, a study compared the effects of slow versus fast visits to a web-based art gallery combined with mindset framing instructions given to individuals (ie, to mindfully engage with art, to engage in social looking, and to engage in curious looking) on outcomes including engagement, meaning, and autonomy satisfaction [36]. The study found no effects, which were attributed to variable selection and the potential impact of unmeasured third factors, such as positive emotion triggered by art engagement [36].

To obtain a more comprehensive picture of the situational factors that affect the positive effects of digital art exposure, this study explores the influence of attachment security or avoidance priming. According to adult attachment theory, repeated interactions allow individuals to develop mental models about close relationships [37]. For instance, secure attachment is linked to the belief that significant others are trustworthy [38]. In comparison, attachment avoidance is linked to the belief that significant others cannot be trusted [39]. The application of priming techniques to trigger mental models of attachment security or avoidance can lead to a temporary enhancement of people’s sense of security or anxiety due to the activation of pre-existing attachment-related memories [40,41]. Thus, attachment security priming can increase individuals’ health and well-being, as the contextual activation of a nurturing attachment figure reduces stress and increases positive mood [42]. For example, attachment security priming can reduce psychological pain (ie, hurt feelings) relative to neutral primes [43]. Additionally, viewing photos of a significant other while receiving painful stimuli reduces pain ratings and pain-related neural activation and augments activity in safety signaling neural regions [44]. Moreover, secure and avoidant attachment primes increase or decrease pain threshold and tolerance relative to a neutral prime, respectively [45]. According to a meta-analysis [46], attachment security priming has larger effect sizes on affect-related outcomes (eg, decreased pain, lower anxiety, and positive affect) relative to its effects on behavior (eg, trust and altruism) and cognition (eg, attitude change, reaction time, and recall). Thus, secure attachment priming should enhance the effects of digital art exposure, whereas avoidant attachment priming should have the opposite effect.

### Objectives and Hypotheses

This study is the first randomized controlled trial to explore the impact of a web-based art museum serious game on pain and social disconnection among individuals living with chronic pain and loneliness, thereby responding to both the global need for more research on how game design and behavior change interventions impact health [47] and the specific need within clinical pain management to develop nonpharmacological self-management treatment approaches to chronic pain [48] that target social disconnection [5,12]. This study tests the joint and separate effects of exposure to digital art and attachment figure priming on pain and social disconnection. The study also examines how enhanced positive feelings about the social world triggered by artwork exposure and frequency of past museum visits mediate and moderate pain and social disconnection effects, respectively. The study’s hypotheses are presented in Multimedia Appendix 1.

## Methods

### Ethical Considerations

The University of California, Davis Institutional Review Board approved this study (IRB# 1811205‐1). All participants were presented with a detailed study description before providing informed consent prior to randomization into the experimental conditions detailed below. The study data are anonymous and deidentified. The data are stored in a password-protected computer with push authentication. Participants were compensated with a US $25 electronic gift card.

### Study Design

This randomized controlled trial used a 2 (digital artwork present and absent) × 2 (secure attachment and avoidant attachment prime) repeated measures factorial web-based experimental design with a hanging control condition. The hanging control group was not exposed to any level of the independent variables and thus represented a strict no-exposure condition [49].

Based on a priori power analysis [50] for an ANOVA (analysis of variance) design with repeated measures within-between interaction and power estimated at 1−β=0.80, Cohen *d*=0.10, it was estimated that the study required 305 participants to have enough statistical power to test its hypotheses. Eligible participants were randomized by StudyPages (Yuzu Labs), a third-party Health Insurance Portability and Accountability Act–compliant clinical trial management platform, using computer-generated blocks of 5 to ensure balanced allocation across study groups. This study was registered on ClinicalTrials.gov (NCT05310747). The hypotheses and data analysis plan were preregistered in the Open Science Framework.

### Recruitment and Sample

StudyPages conducted a social media campaign to recruit participants for this study. Older adults living in the United States were targeted with advertisements on Facebook and Instagram inviting them to participate. Recruitment occurred between October 9, 2022, and January 3, 2023. A total of 1789 participants were invited to the study: 398 accessed the study from StudyPages’ Facebook and Instagram social media campaign and 1391 accessed the study from direct and unknown sources. Of the participants who accessed the study from known sources, 65 were linked to StudyPages via SMS share, 28 via email share, 7 through Facebook share, and 1 via Twitter share.

All invited participants were first directed to StudyPages’ website to respond to a 5-question eligibility screener. The inclusion criteria stipulated (1) English language proficiency, (2) adults ≥18 years of age, (3) chronic moderate to severe pain (≥6 months in duration and ≥4 in response to the question, “on average this week my pain intensity has been?” on a 0‐10 numeric rating scale) [51], (4) loneliness (≥4 on the 3-item Loneliness Scale) [52], and (5) ownership of an electronic device with internet connection. Individuals were excluded from this study if they had dementia or were unable to consent. Participants were not screened for analgesic use. In total, 382 participants failed a single or more criterion of the eligibility screener and were thus excluded from the study. Participants’ self-reports were used for the eligibility screener and were not independently verified. All participants who passed the eligibility screener were enrolled into the study. Additional information can be found in Multimedia Appendices 2 and 3.

Bot attacks occurred throughout data collection. Several safety measures were used to eliminate any fraudulent data from the final analysis. The clinical trial recruitment platform used bot detection techniques, such as eliminating data that resulted from large numbers of signups within a short period of time and checking for fraudulent or duplicate IP addresses. Additionally, the third author (WW) filtered all responses to ensure the validity of all data, checking for duplicate responses and invalid text entries.

### Intervention Design and Procedures

The web-based art museum gallery ran on Unity (version 2020.3.21; Unity Technologies), a digital platform for creating real-time 3D digital games. Participants used their computer’s browser to complete all study surveys and explore a custom-designed web-based serious game simulating a 3D art museum gallery for 10 minutes. Participants filled out a pretest survey measuring pain and social disconnection and then responded to 3 writing prompts.

To prime secure or avoidant attachment, participants provided the initials for a close person that had made them feel socially connected or disconnected (ie, loved or valued and unloved and undervalued) [42,53]. Following this, they were given 5 minutes to provide a minimum of 80 words (with a maximum of 500 words) in response to “Try to get a visual image in your mind of this person. What is or was it like being with this person? What is or was it about this person that made you feel seen, heard, and valued?” Participants then wrote about a specific occasion or anecdote in which the person in question had said or done something that made the participant feel loved or unloved, cared or uncared for, and valued or undervalued. For the second and third prompts, participants were able to submit their responses after providing at least 80 words or the onscreen timer ran out. Participants then engaged with a tutorial to learn how to move and explore objects of art in the gallery using their keyboard and mouse or trackpad. The tutorial lasted until participants completed a set of 8 simple movement and clicking instructions. The tutorial could be completed in a minimum of 30 seconds. Thus, depending on writing speed and verbosity, participants took 5‐10 minutes to complete attachment priming manipulation and then enter the web-based museum.

**Figure 1.**
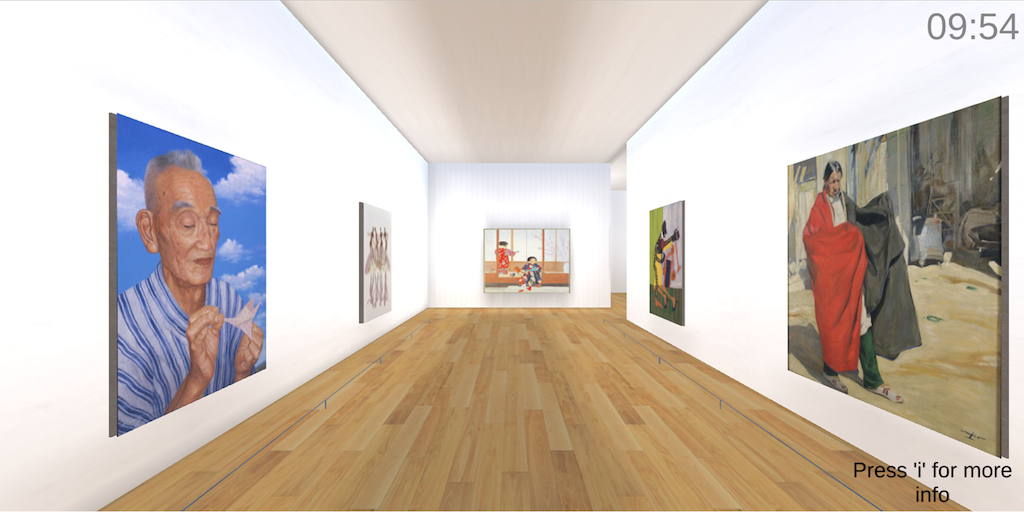
Web-based art museum (artwork present condition).

For the artwork present condition, the principal investigators (JP and IK) selected 18 artworks from the digitized Google Arts & Culture collection pretested to elicit social connection and positive affect, including intimate connection with friends or family, calmness, wonder and awe, love, and liveliness [54]. The selection process attempted to sample artwork from different continents. The gallery included artworks by Myoe Thant Oung (*Living Under Belief 1 and 2*), Vera Bocaiuva Mindlin (*Figuras*), Shoko Uemura (*A Warm Winter Day*), William H Johnson (*Jitterbugs II*), Walter Ufer (*Jim*), Hovhannes Aivazovsky (*The Ninth Wave*), Martin Ron (*Pedro Luján and His Dog*), Georges Seurat (*A Sunday on La Grande Jatte*), Filipp Malyavin (*Whirlwind*), Lee Sangwon (*Aquarium*), Vincent Van Gogh (*Portrait of Joseph Roulin*), Claude Monet (*Woman With a Parasol*), Prateep Kochabua (*Luang Ta Ma*), Diego Rivera (*The Grinder*), Martin Djukin (*Return From the Field*), Boris Kustodiev (*Shrovetide*), and Joaquín Sorolla (*After Bathing*). This approach is consistent with Cotter et al [36], who exposed participants to web-based art galleries with 30 artworks each. For the artwork absent condition, the gallery was the same except the 18 paintings were removed. Figures1  2 depict the artwork present versus absent conditions.

**Figure 2.**
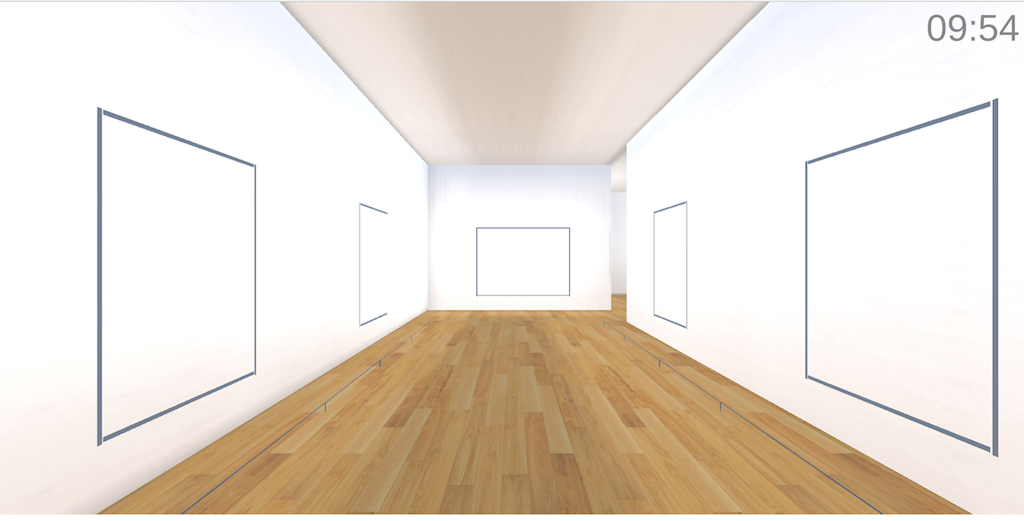
Web-based art museum (artwork absent condition).

Participants in the hanging control group filled out the preintervention survey and then waited for 10 minutes before completing the postintervention survey.

### Dependent, Mediator, and Moderator Variables

This study’s dependent variables, perceived pain and social disconnection, were measured immediately before and after the assigned intervention. We measured several dimensions of pain with pre- and posttest single-item 0‐10 Likert numeric rating scales: pain intensity (“What is your pain intensity right now?”), pain unpleasantness (“How unpleasant is your pain right now?”), general pain interference (“What number best describes how, at the moment, pain has interfered with your general activity?”), and pain enjoyment interference (“What number best describes how, at the moment, pain has interfered with your enjoyment of life?”) [55]. These items showed significant positive correlations (*r*_pretest_(310)=0.56, 0.61, 0.77, 0.83; *P*=.001 and *r*_posttest_(308)=0.74, 0.77, 0.79, 0.91; *P*=.001, respectively). An exploratory factor analysis indicated that the scales resolved in a single pain factor that explained 85.6% of variance in the responses. Thus, the pain-related items were averaged into 2 variables that represented pre- (mean 7.00, SD 1.63; Cronbach α=0.89) and posttest perceived pain (mean 5.96, SD 2.13; Cronbach α=0.94).

Perceived social disconnection was measured using a 12-item social disconnection scale [56] on a 5-point Likert scale (1=not at all and 5=very much). Sample items include “I feel disconnected from others” and “I feel alone.” Social disconnection scale scores were clustered around the average, and both pre- and posttest scores demonstrated good reliability (mean 3.02, SD 0.41; Cronbach α=0.89) and posttest (mean 2.96, SD 0.89; Cronbach α=0.92).

Feelings about the social world triggered by the artwork were measured immediately after the intervention using the average score across 4 items presented as 1‐7 Likert-type scale (1=strongly disagree and 7=strongly agree). Items included “The artwork in the museum made me feel (negative-positive about the social world, disconnected-connected to the social world, excluded-included from the social world, and negative-positive about my social relationships).” The scale was reliable (mean 4.80, SD 1.36; Cronbach α=0.91). This variable was used as a statistical mediator to examine how positive feelings triggered by the artwork would indirectly influence the direct link between the manipulations and outcomes such as pain and social disconnection.

The frequency of museum visits was measured with a single item, which asked participants “How often did you visit an art museum last year?” The response options were none, 1 time, 2 times, 3 times, and more than 4 times (mean 1.94, SD 1.20). This variable was used to examine how pre-existing levels of engagement with museums would moderate an indirect link between artwork and attachment priming manipulations and pain and social disconnection.

### Data Analysis

The data were analyzed with repeated measures ANOVAs to account for within and between subjects effects before and after the experimental manipulations [51]. We used Bonferroni-corrected tests for multiple comparisons to avoid spurious results. The data were also analyzed with the Process Macro Model 59 for multicategorical moderated mediation tests to account for the effect of a single moderator on all 3 mediation pathways [52]. Six multicategorical moderated mediation models were constructed to assess the main and interaction effects of the artwork and priming manipulations. The hanging control condition was set as the reference group. Postexperiment pain scores and social disconnection scores were the outcome variables. Positive feelings about the social world prompted by the artwork was the mediator variable. The frequency of museum visits was used as the moderator variable. The mediator and moderator variables were mean centered. The indirect effects of the experimental conditions were bootstrapped with 5000 samples with replacement. All tests were carried out with SPSS (IBM Corp).

## Results

### Sample

**Figure 3.**
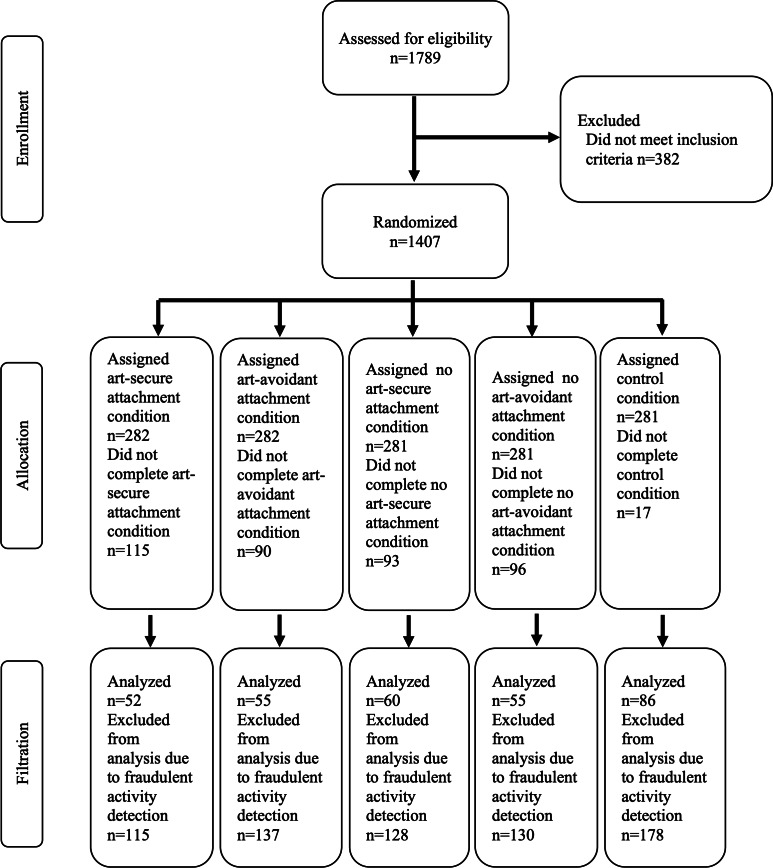
CONSORT (Consolidated Standards of Reporting Trials) diagram.

In total, 1407 participants were randomized. Among them, 411 participants did not complete their randomly assigned condition, and cases were removed following bot activity. The study enrolled 308 participants. Participants’ mean age was 42.78 (SD 13.11; range 18-76) years. Among 308 participants, 185 (60.06%) were identified as women, 113 (36.69%) as men, 9 (2.92%) as nonbinary, and 1 (0.32%) selected other. A total of 67 of 308 (21.75%) participants were African American, 6 (1.95%) were American Indian or Alaska Native, 17 (5.52%) were Asian, 212 (68.83%) participants were White, 3 (.94%) selected unknown race, and 3 (.94%) preferred not to answer. Of the participants who selected White, 14(6.6%) were Hispanic or Latino. Two participants were removed from analysis through the listwise deletion because of missing data. Thus, the analysis below is based on 308 participants. Figure 3 summarizes participant enrollment, allocation, and analysis.

### Main Effects of Art Exposure on Pain

There was a significant pre- to postintervention difference between the artwork present and absent conditions (*F*_2,305_=14.90; *P*=.001; *r*=0.22). As expected, there was no reliable pre- to posttest pain difference for the hanging control group (*P*=.07). Posttest pain was lower than pretest pain for the artwork present (*P*=.001) and absent conditions (*P*=.001). Relative to the control group, the artwork present (*P=*.001) and absent conditions (*P*=.01) had decreased posttest pain scores. There were no posttest pain differences between the artwork present and absent conditions (*P*=.21). See Table 1 for descriptive statistics.

**Table 1.** Pain scores (n=308)^a^.

	Pretest pain, mean (SD)		Posttest pain, mean (SD)		Number of participants, n (%)
**Conditions**					
Artwork	6.96 (1.63)		5.39 (2.11)		107 (34.7)
No artwork	6.99 (1.64)		5.89 (2.15)		115 (37.3)
Control	7.06 (1.65)		6.74 (1.88)		86 (27.9)
Total	7.00 (1.63)		5.96 (2.13)		308 (100)
**Conditions**					
Secure attachment	7.16 (1.55)		5.66 (2.23)		112 (36.3)
Avoidant attachment	6.78 (1.69)		5.64 (2.06)		110 (35.7)
Control	7.06 (1.65)		6.74 (1.88)		86 (27.9)
Total	7.00 (1.63)		5.96 (2.13)		308 (100)
**Conditions**					
Art-secure attachment	7.08 (1.73)		5.28 (2.34)		52 (16.8)
Art-avoidant attachment	6.84 (1.53)		5.50 (1.88)		55 (17.8)
No art-secure attachment	7.23 (1.40)		5.99 (2.09)		60 (19.4)
No art-avoidant attachment	6.72 (1.84)		5.79 (2.23)		55 (17.8)
Control	7.06 (1.65)		6.74 (1.88)		86 (27.9)
Total	7.00 (1.63)		5.96 (2.13)		308 (100)

a Pain score range 0-10, where 0=no pain and 10=worst pain imaginable.

### Main Effects of Art Exposure on Social Disconnection

Though there were no reliable pre- to postintervention differences between the artwork present and absent conditions (*F*_2,305_=1.91; *P*=.15; *r*=0.08), social disconnection decreased from pre- to posttest for the artwork present condition (*P*=.04). There were no pre- to posttest social disconnection differences for the artwork absent (*P*=.51) and control conditions (*P*=.45). Posttest social disconnection was lower in the artwork present condition relative to the control group (*P*=.02). Posttest social disconnection scores did not differ between the artwork present and absent conditions (*P*=.14). There were no posttest social disconnection differences between the no artwork condition and the control group (*P*=.96). See Table 2 for details.

**Table 2.** Social disconnection scores (n=308)^a^.

	Pretest social disconnection, mean (SD)		Posttest social disconnection, mean (SD)		Number of participants, n (%)
**Conditions**					
Artwork	2.95 (0.47)		2.77 (0.81)		107 (34.7)
No artwork	3.06 (0.38)		3.01 (0.93)		115 (37.3)
Control	3.06 (0.38)		3.13 (0.89)		86 (27.9)
Total	3.02 (0.42)		2.96 (0.89)		308 (100)
**Conditions**					
Secure attachment	3.06 (0.44)		2.80 (0.91)		112 (36.3)
Avoidant attachment	2.95 (0.41)		2.99 (0.84)		110 (35.7)
Control	3.06 (0.38)		3.13 (0.89)		86 (27.9)
Total	3.02 (0.42)		2.96 (0.89)		308 (100)
**Conditions**					
Art-secure attachment	3.00 (0.50)		2.69 (0.82)		52 (16.8)
Art-avoidant attachment	2.91 (0.43)		2.85 (0.79)		55 (17.8)
No art-secure attachment	3.12 (0.38)		2.89 (0.98)		60 (19.4)
No art-avoidant attachment	3.00 (0.38)		3.13 (0.87)		55 (17.8)
Control	3.06 (0.38)		3.13 (0.89)		86 (27.9)
Total	3.02 (0.42)		2.96 (0.89)		308 (100)

a Social disconnection score range 1-5, where 1=not at all and 5=very much.

### Main Effects of Attachment Priming on Pain

There was a reliable pre- to posttest difference between the attachment security priming conditions (*F*_2,305_=13.80; *P*=.001; *r*=0.21). More specifically, there was a pre- to posttest pain decrease for the secure (*P*=.001) and avoidant priming conditions (*P=*.001). There were no pre- to posttest pain differences for the control group (*P*=.07). The secure (*P*=*.*001) and avoidant attachment conditions (*P*=.001) had decreased posttest pain scores relative to the control group. There was no posttest pain difference between the secure and avoidant attachment priming conditions (*P*>.99).

### Main Effects of Attachment Priming on Social Disconnection

There was a significant pre- to posttest social disconnection score decrease for the secure and avoidant attachment priming conditions (*F*_2,305_=4.66; *P*=.01; *r*=0.12). Closer inspection revealed a pre- to posttest social disconnection decrease for the secure attachment condition (*P=.*002) but no pre- to posttest differences for the avoidant priming (*P*=.65) and control conditions (*P*=*.*45). There were no postsocial disconnection differences between the secure and avoidant attachment conditions (*P*=*.*30) and no posttest social disconnection differences between the avoidant attachment condition and the control group (*P*=.81), though posttest social disconnection was lower for the secure attachment condition relative to the control group (*P*=.03).

### Interaction Effects

#### Pain

There was a reliable pre- to posttest difference between the 5 experimental conditions (*F*_4, 303_=8.30; *P*=.001; *r*=0.16). We found a pre- to posttest pain decrease for the artwork-secure attachment (*P*=.001), artwork-avoidant attachment (*P*=.001), no artwork-secure attachment (*P*=.001), and no artwork-avoidant attachment conditions (*P*=.001). There were no significant pre- to posttest pain differences for the control group (*P*=.06). Compared with the control group, the artwork-secure (*P*=.001) and artwork-avoidant attachment conditions (*P*=.006) had lower posttest pain scores. The no artwork-avoidant attachment (*P*=*.*08) and no artwork-secure attachment (*P*=.32) showed no posttest pain differences compared with the control group.

#### Social Disconnection

There was a reliable pre- to postscore difference between the 5 experimental conditions (*F*_4,303_=2.70; *P*=.03; *r*=0.09). The artwork-secure attachment (*P*=.01) and the no artwork-secure attachment conditions (*P*=.05) had lower postrelative to pretest social disconnection scores. Posttest social disconnection was lower for the artwork-secure attachment condition compared with the control group (*P*=*.*04). No posttest social disconnection differences were found between the artwork-avoidant attachment (*P*=.66), no artwork-secure attachment (*P*>.99), and no artwork-avoidant attachment conditions (*P*>.99) compared with the control group.

### Mediation Effects of Feelings About the Social World Triggered by Artwork Exposure and Moderation Analyses of Frequency of Museum Visits on Pain and Social Disconnection

#### Artwork Exposure Effects

Relative to the control group, the artwork present condition reported more positive feelings about the social world triggered by the artworks (*B*=.69; SE 0.14; *t*_308_=3.63; *P*=.001; 95% CI 0.31-1.06). There was no difference between the no artwork condition and the control group regarding positive feelings (*B*=.01; SE 0.19; *t*_308_=0.03; *P*=.98; 95% CI −0.03 to 0.42).

Additionally, increased positive feelings were linked to decreased posttest pain (*B*=−.53; SE 0.09; *t*_308_=−6.17; *P*=.001; 95% CI −0.70 to −0.36) and lower posttest social disconnection (*B*=−.25; SE 0.04; *t*_308_=−7.17; *P*=.001; 95% CI −0.32 to −0.18). Increased positive feelings were not linked to more frequent museum visits (*B*=.20; SE 0.11; *t*_308_=1.71; *P*=.09; 95% CI −0.03 to 0.42) and decreased social disconnection (*B*=−0.12; SE 0.07; *t*_308_=−1.67; *P*=.10; 95% CI −0.26 to 0.02).

Participants in the artwork present condition at average (*B*=−.98; SE 0.29; *t*_308_=−3.38; *P*=.001; 95% CI −1.56 to −0.41) and above average frequency of museum visits (*B*=−1.36; SE 0.40; *t*_308_=−3.40; *P*=.001; 95% CI −2.15 to −0.57) reported decreased posttest pain. Participants in the artwork absent condition at average (*B*=−.81; SE 0.28; *t*_308_=−2.89; *P*=.001; 95% CI −1.36 to −0.26) and above average museum visit frequency (*B*=−.99; SE 0.38; *t*_308_=−2.60; *P*=.01; 95% CI −1.76 to −0.24) had decreased posttest pain.

The average frequency of museum visits moderated the indirect effect of positive feelings on the link between artwork exposure and pain. Participants in the artwork present condition at above average (*B*=−.37; SE 0.15; 95% CI −0.70 to −0.10), average (*B*=−.37; SE 0.07; 95% CI −0.61 to −0.16), and below average museum visit frequency showed decreased posttest pain (*B*=−.37; SE 0.09; 95% CI −0.72 to −0.10).

Moreover, the mediation effect of positive feelings on the link between artwork exposure and social disconnection was moderated by the average frequency of museum visits. Participants below the average of frequency of museum visits in the artwork present condition reported decreased posttest social disconnection (*B*=−.32; SE 0.15; *t*_308_=−2.12; *P*=.03; 95% CI −0.62 to −0.02). Participants in the artwork present condition at above average (*B*=−.22; SE 0.08; 95% CI −0.38 to −0.06), average (*B*=−.17; SE 0.05; 95% CI −0.28 to −0.08), and below the average frequency of museum visits also showed decreased posttest social disconnection (*B*=−.14; SE 0.06; 95% CI −0.27 to −0.04).

#### Attachment Priming Effects

There was no difference between the control group and the secure attachment priming condition regarding positive feelings about the social world (*B*=.34; SE 0.19; *t*_308_=1.76; *P*=.08; 95% CI −0.04 to 0.71). There was also no difference between the control group and the avoidant attachment priming condition regarding positive feelings (*B*=.31; SE 0.19; *t*_308_=1.60; *P*=.11; 95% CI −0.07 to 0.69).

The manipulations operated equally among participants at multiple levels of museum visit frequency. Participants in the secure attachment condition below average (*B*=−.73; SE 0.37; *t*_308_=−1.96; *P*=.05; 95% CI −1.46 to −0.01), at average (*B*=−.88; SE 0.28; *t*_308_=−3.08; *P*=.001; 95% CI −1.44 to −0.32), and above the average of frequency of museum visits reported decreased posttest pain (*B*=−1.07; SE 0.39; *t*_308_=−2.75; *P*=.006; 95% CI −1.83 to −0.30).

Participants in the avoidant attachment condition at average (*B*=−.91; SE 0.29; *t*_308_=−3.18; *P*=.001; 95% CI −1.47 to −0.35) and above the average of frequency of museum visits reported decreased posttest pain (*B*=−1.27; SE 0.40; *t*_308_=−3.21; *P*=.001; 95% CI −2.05 to −0.49). Secure attachment priming reduced posttest social disconnection compared with the control condition (*B*=−.27; SE 0.11; *t*_308_=−2.33; *P*=.02; 95% CI −0.49 to −0.04).

#### Interaction Effects

Participants in the artwork-secure attachment condition had increased positive feelings about the social world relative to the control condition (*B*=.80; SE 0.23; *t*_308_=3.47; *P*=.001; 95% CI 0.34-1.25). In addition, participants in the artwork-avoidant attachment condition had increased positive feelings compared with the control condition (*B*=.57; SE 0.23; *t*_308_=2.52; *P*=.01; 95% CI 0.12-1.02). However, compared with the control condition, the no artwork-secure attachment condition and the no artwork-avoidant attachment condition did not influence positive feelings (*B*=−.06; SE 0.22; *t*_308_=−0.28; *P*=.78; 95% CI −0.49 to 0.37 and *B*=.05; SE 0.23; *t*_308_=0.22; *P*=.83; 95% CI −0.39 to 0.49).

Participants in the artwork-secure attachment condition above the average (*B*=−.36; SE 0.20; 95% CI −0.80 to −0.02), at average level (*B*=−.43; SE 0.14; 95% CI −0.74 to −0.18), and below the average of frequency of museum visits showed decreased posttest pain (*B*=−.51; SE 0.19; 95% CI −0.92 to −0.17). In addition, participants in the artwork-avoidant attachment condition below the average and those at average frequency of museum visits had decreased posttest pain (*B=*−.35; SE 0.17; 95% CI −0.71 to −0.06 and *B*=−.31; SE 0.13; 95% CI −0.57 to −0.07, respectively).

The average frequency of museum visits moderated the indirect effect of positive feelings on the link between artwork-secure attachment manipulations and social disconnection. Participants above the average (*B*=−.14; SE 0.08; 95% CI −0.32 to −0.01), at the average (*B*=−.20; SE 0.06; 95% CI −0.33 to −0.08), and below the average frequency of museum visits showed lower posttest social disconnection (*B*=−.29; SE 0.09; 95% CI −0.48 to −0.12).

The indirect effect of positive feelings on the link between artwork-avoidant attachment manipulations and social disconnection was moderated by the average frequency of museum visits. Participants below the average frequency of museum visits (*B*=−.09; SE 0.04; 95% CI −0.18 to −0.01) reported decreased posttest social disconnection. See Table 3 for additional details.

**Table 3.** Scores for positive feelings about the social world triggered by artwork and frequency of museum visits in the last year (N=308)^a^.

Condition			Score, mean (SD)	Number of participants, n (%)
**Positive feelings about the social world triggered by artwork**				
	**Artwork**			
		Secure attachment	5.36 (1.33)	52 (16.8)
		Avoidant attachment	5.12 (1.23)	55 (17.8)
	**No artwork**			
		Secure attachment	4.51 (1.48)	60 (19.4)
		Avoidant attachment	4.60 (1.38)	56 (18.1)
	**Control**		4.60 (1.22)	86 (27.8)
	**Attachment priming**			
		Secure attachment	4.91 (1.47)	112 (36.2)
		Avoidant attachment	4.86 (1.33)	111 (35.9)
**Frequency of museum visits in the last year**				
	**Artwork**			
		Secure attachment	1.94 (1.16)	52 (16.8)
		Avoidant attachment	1.80 (1.24)	55 (17.8)
	**No artwork**			
		Secure attachment	1.97 (1.22)	60 (19.4)
		Avoidant attachment	1.79 (1.14)	56 (18.1)
	**Control**		2.10 (1.23)	86 (27.8)
	**Attachment priming**			
		Secure attachment	1.96 (1.19)	112 (36.2)
		Avoidant attachment	1.79 (1.18)	111 (35.9)

a Frequency of museum visits in the last year score range 1-5, where 1=none and 5=more than 3 times.

## Discussion

### Principal Findings

This randomized controlled trial found evidence that digital art exposure using a serious game simulating a visit to an art museum reduced pain and social disconnection among individuals experiencing chronic pain and loneliness. These findings were especially relevant, considering how similar interventions had not examined the effects of digital art engagement on pain [31,32].

In support of the prediction that digital art exposure exerts its salutogenic effects by increasing positive affect [24,28,30], we found that more positive feelings about the social world triggered by the artworks mediated the effects of artwork exposure on pain and social disconnection.

### Comparisons to Prior Work and Future Directions

The findings resonated with how digital art engagement decreased negative mood [31] and increased feelings of pleasure [32]. Future research should compare the effects of exposure length and temporal contiguity between digital art exposure and data collection. This study exposed participants to a gallery with 18 artworks for 10 minutes, whereas Trupp et al [31,32] exposed participants to a single artwork for 1.5 to 2 minutes. Outcome measures remained unaffected after four 15-minute gallery visits when the data were collected 1 week after exposure [36], but reliable effects of digital art exposure were obtained when measured right after a digital gallery visit [57].

The selected artwork was effective at bolstering positive social feelings. Though engagement with the arts in the physical world can reduce loneliness [11,35], by showing that short-term exposure to artwork in a web-based serious game alleviated pain and social disconnection, our study stresses how experiences with digital art can have positive effects even when people are isolated from others and did not physically visit an exhibition.

In addition, the effect of positive feelings triggered by artwork exposure on decreasing pain and social disconnection was effective across individuals who engage with museums at low, average, and high frequency. Individual differences still deserve further exploration. In prior work, individuals’ aesthetic responsiveness increased liking and meaning triggered by a digital Monet painting, which in turn augmented positive mood, decreased negative mood, and alleviated state anxiety [32]. It is possible that ingrained aesthetic sensitivities are more predictive than actual physical visits to museums.

The artwork and the secure attachment prime had only a few direct effects, implying that feelings about the social world triggered by artwork exposure significantly explained the observed palliative effects on pain and social disconnection. In addition, attachment security priming manipulations did not influence positive feelings about the social world. It is possible that priming and mindset framing techniques do not reliably enhance well-being above and beyond digital art exposure. In prior work, there were no differences between visitors of a digital art gallery instructed to mindfully engage with artwork, relate the art to important personal relationships, or consider the art and the artist [36]. Future research should test whether exposure to digital art overrides the effect of an observer’s situational mindset.

Contrary to our predictions, all conditions combining the presence or absence of artwork with attachment security priming reliably reduced pain compared with a control condition. These results on their own could have implied placebo and Hawthorne effects at play. Such effects have appeared in clinical trials studying pain [33] and in a recent test of the effects of digital art exposure [31,32]. Though placebo and Hawthorne effects may have been at play, the strongest palliative effects on pain and social disconnection were driven by feelings about the social world triggered by artwork exposure. Furthermore, the no artwork exposure condition did not affect positive feelings about the social world and thus, as one would expect, positive feelings did not mediate the effects on pain and social disconnection in this condition. The control group did not experience significant pre- to posttest changes, even though participants in this condition underwent the same procedure as participants in the remaining conditions save for the manipulations. This further disconfirmed the placebo and Hawthorne effects.

### Limitations

Participants were recruited using a social media campaign. Thus, non–social media users were not represented in the sample. Bot attacks affected the integrity of random allocation.

The web-based serious game ran on a desktop browser so only individuals who owned PCs had access to the study. Future research should replicate these findings using mobile phone, and VR game builds to account for how different gaming platforms may influence the results. For instance, digital art exposure on smartphone had smaller effects relative to laptop or desktop computer viewing [32]. This effect was driven by a decrease in liking of art when viewed through smartphones relative to desktops and laptops [32]. The results of this study represent single exposure effects so future research should investigate potential longitudinal effects and the influence of repeated web-based museum visits. Additionally, though the social media campaign targeted a diverse pool of potential participants, the eligible sample overrepresented middle-aged White women relative to other social groups, which may affect its generalizability. The study only focused on self-reported data. The data were collected through winter months and, thus, seasonality may have affected the responses.

### Conclusions

Playing a web-based serious game that allowed individuals living with chronic pain and loneliness to visit a digital art museum reliably decreased pain and social disconnection. This clinical trial highlighted how exposure to digital art reduces the burden of chronic pain and social disconnection chiefly through elevating positive social affect. As such, this study contributed to the evidence that VR is an effective nonpharmacological self-management treatment approach for chronic pain management.

## Supplementary material

10.2196/52294 Multimedia Appendix 1 Hypotheses.

10.2196/52294 Multimedia Appendix 2 Social media campaign.

10.2196/52294 Multimedia Appendix 3 Sample characteristics.

10.2196/52294 Checklist 1 CONSORT-eHEALTH checklist (V 1.6.1).

## Abbreviations

ANOVA: analysis of variance
VR: virtual reality
