# Supplementary material for: Using Digital Art and Attachment Priming in a Web-Based Serious Game to Reduce Pain and Social Disconnection in Individuals With Chronic Pain and Loneliness: Randomized Controlled Trial
Source: JMIR Serious Games. 2024 Nov 27;12:e52294. doi: 10.2196/52294 (PMC11612526; doi:10.2196/52294)
Supplement: Multimedia Appendix 1 [file games-v12-e52294-s001.docx]

# Can Virtual Art and Attachment Priming Decrease Pain and Social Disconnection Among Individuals Living with Chronic Pain and Loneliness? A Randomized Controlled Trial Using a Non-Immersive Virtual Reality Serious Game

The study tested the following hypotheses:

Hypothesis 1: Exposure to virtual artwork will decrease (1a) pain and (1b) perceived social disconnection relative to individuals in a no artwork condition.

Hypothesis 2: Exposure to virtual artwork will decrease (2a) pain and (2b) perceived social disconnection relative to individuals in a control condition.

Hypothesis 3: Experiencing a secure attachment prime prior to exposure to a virtual artwork condition will decrease (3a) pain and (3b) perceived social disconnection compared with experiencing an avoidant attachment prime prior to exposure to a virtual artwork condition.

Hypothesis 4: Experiencing a secure attachment prime prior to exposure to a virtual artwork condition will decrease (4a) pain and (4b) perceived social disconnection compared with individuals in a control condition.

Hypothesis 5: Experiencing a secure attachment prime prior to exposure to a virtual artwork condition will decrease (5a) pain and (5b) perceived social disconnection compared with experiencing an avoidant attachment prime prior to exposure to a no artwork condition.

Hypothesis 6: Feelings triggered about the social world by the artwork in the museum will mediate the effects of secure attachment or avoidant attachment primes and artwork presence or absence on (6a) pain and (6b) perceived social disconnection.

Hypothesis 7: Frequency of visits to real museums will moderate the effects of secure attachment or avoidant attachment primes and artwork presence or absence on (7a) pain (7b) perceived social disconnection.
