# Supplementary material for: Using Digital Art and Attachment Priming in a Web-Based Serious Game to Reduce Pain and Social Disconnection in Individuals With Chronic Pain and Loneliness: Randomized Controlled Trial
Source: JMIR Serious Games. 2024 Nov 27;12:e52294. doi: 10.2196/52294 (PMC11612526; doi:10.2196/52294)
Supplement: Multimedia Appendix 2 [file games-v12-e52294-s002.pdf]

StudyPages Ad Campaign Creative

Study Title: Virtual museum study

1. Ad Copy

Campaign Type: Facebook, Instagram

Headlines	• Chronic pain x Virtual museum experience study<br>• Is loneliness increasing chronic pain?<br>• Can a museum experience improve pain and loneliness?<br>• Virtual museum study for people with chronic pain
Ad text	• UC Davis is looking for people with chronic pain who feel lonely to participate in virtual museum-based programs such as virtual tour/virtual meditation at a museum. Participate in a research study!<br><br>• UC Davis researchers are looking for people with chronic pain who feel lonely. We hope to learn if virtual museum engagement can decrease feelings of social disconnection and the unpleasantness of chronic pain. Click to see the details or signup.<br><br>• Have chronic pain? Missing museum visits? Take part in an online museum research study. We hope to learn if virtual museum engagement can decrease feelings of social disconnection and the unpleasantness of chronic pain. Click to learn more or signup.<br><br>• Humans are social beings. Is social disconnection increasing chronic pain? Can participating in virtual museum programs help people with chronic pain feel more socially connected and reduce pain? Help UC Davis researchers find out. Participate in a research study.
Optional Description	• Participate in an at-home research study<br>• Click to learn more and take a survey to see if you may qualify

2. Images for ad (The images and text will be combined to make FB ads)

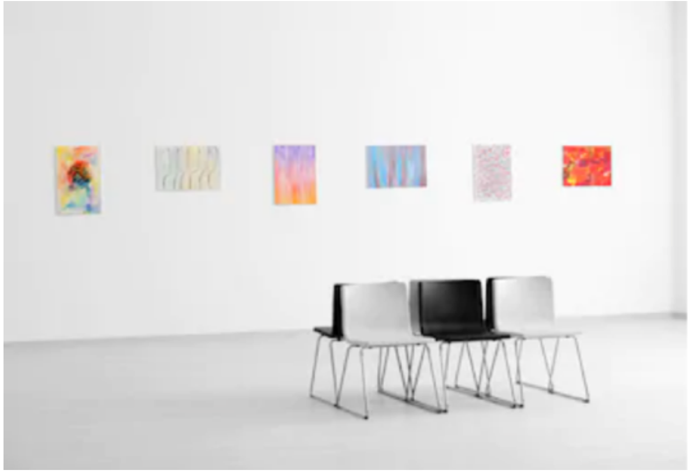

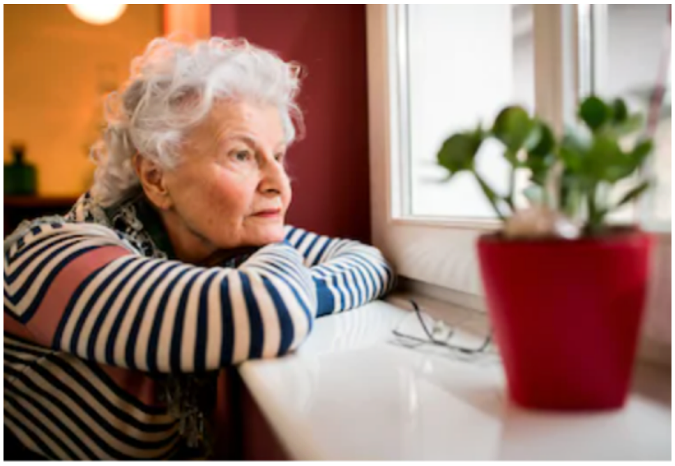

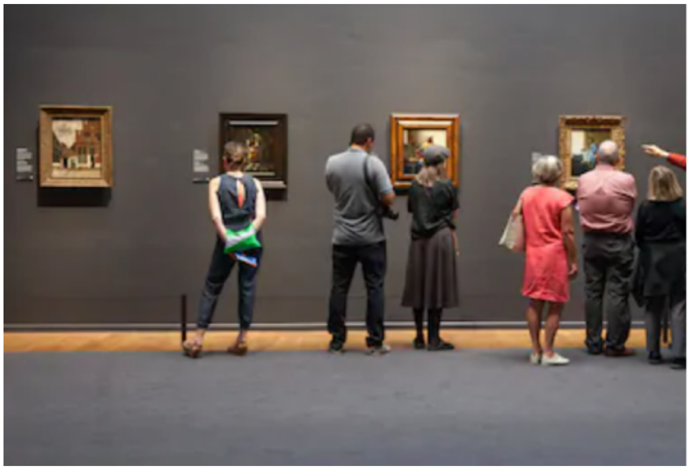

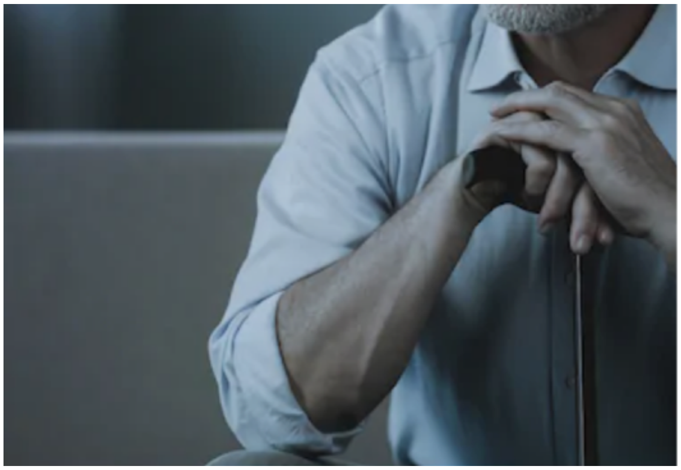

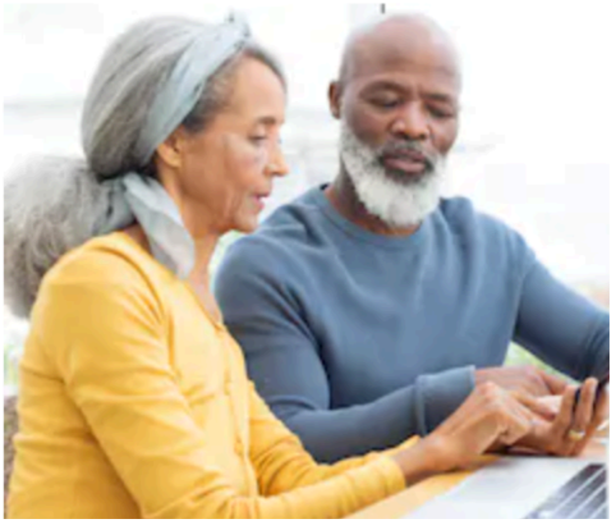

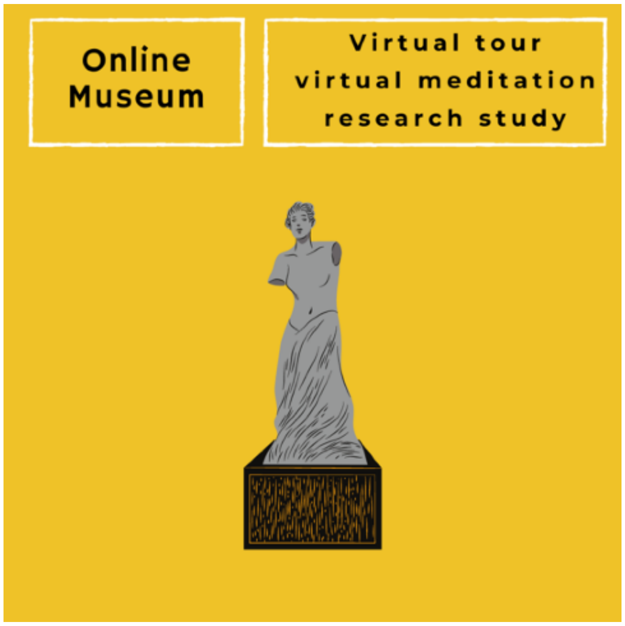

3. Facebook ads link to this website

StudyPages

At-home study of virtual museum experiences to reduce social isolation and pain

UC Davis Health

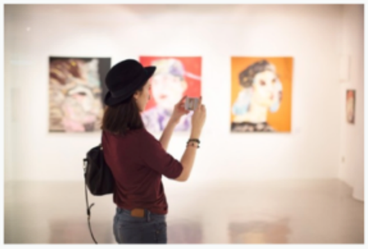

UCDAVIS HEALTH

"Can virtual museum programs help individuals with chronic pain feel more socially connected? Help us find out!"

I'm Interested in this study

Share study

Age: 18+

Gender: Any

Healthy Volunteers: No

Keywords: chronic pain, social isolation, virtual programs

Type: Other Study

Target: 64 Participants

Investigator:

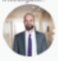

Ian Koebner, Ph.D., M.Sc., M.A.O.M., L.Ac., Assistant Professor
UC Davis Medical Center
View profile

Description

This research study is evaluating virtual museum-based programs to address loneliness and social isolation among individuals with chronic pain.

Over 100 million American adults suffer from chronic pain. This is more than those with heart disease, cancer, and diabetes combined.

Chronic pain is complex. It affects the body, the mind, and social interactions. Previous studies show that social disconnection can cause pain to feel more intense. Also, they show that social connection can help decrease the intensity of pain.

Virtual museum experiences may be able to reduce isolation and give people a sense of social connection. We hope to learn if virtual museum engagement can decrease feelings of social disconnection and the unpleasantness of chronic pain in individuals with chronic pain.

This study requires

If you agree to take part in this research, you will randomly assigned to one of four groups:

- a usual care waitlist control group,
- a virtual museum tour - Art RX,
- a virtual meditation program at the museum - Artful Meditation, or
- both the virtual Art Rx and Artful Meditation programs.

You will be asked to take a survey about your experiences. Surveys will take approximately 20 minutes to complete. They will be administered before, after and at 3 month follow up to the assigned intervention.

You may also be asked to take part in an interview. You will be asked questions about your experiences with museums and arts spaces, the impact these experiences may have had on your health, your health in general, and your relationships. It will take an hour or less to complete the interview.

The interview will be audiotaped and transcribed, but your name will not be included on the transcription.

Who can participate

Inclusion Criteria:

1. 18 years of age or older
2. English speaking
3. Chronic pain (6 months or longer)
4. Moderate pain or greater
5. Moderately lonely or greater

Exclusion Criteria:

1. Participated in an Art Rx tour
2. Participated in an Artful Meditation program
3. Dementia or Alzheimer's Disease
4. Unable to complete surveys

Benefits and risks of participating

BENEFITS:

We cannot promise any benefits to you as a result of taking part in this research study.

RISKS:

The Principal Investigator or a Research Assistant will discuss the risks associated with taking part in this research study.

Compensation

Participants will be entered into a drawing for a $50 gift card.

Resources

Study ID

UC Davis IRB: 1415639
ClinicalTrials.gov: NCT04091893

Schedule

Study duration and period

Each survey will take approximately 20 minutes to complete. It will take about an hour to complete the interview.

Recruitment period

From Oct. 16, 2019

Location

Crocker Art Museum
216 O Street
Sacramento, CA 95814

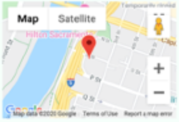

Contact

Ruchi Rawal

ucdhartrx@gmail.com

(916) 619-3383

Research Topic

Conditions:

- chronic pain
- social isolation

Have any questions or want to learn more? Leave your contact details below and the research team will reach out to you.

I'm Interested

Privacy Policy Terms of Use

© 2020 StudyPages by Yuzu Labs PBC

6. Signup form

Signup form:

- Body text : Enter your info and the study team will contact you soon! Your data is securely stored and only shared with the research team
- Entry form: Name, Email, Phone
- Check box: Notify me of future research (opt-out anytime)
- Submit button

7. Study Voicemail

Website visitors who call the contact phone number may hear a voicemail outside of office hours or if no one from the team answers. The transcription of the voicemail message is:

"Thank you for your interest in our virtual museum study. Please leave your name and best contact and we will return your message as soon as we can."

8. Auto-reply text message

Website visitors who sign up for the study will receive a text message (only when signing up with a mobile phone number) that reads:

"Thank you for your interest in our virtual museum study! Please let us know when it's a good time to give you a call. "
