# Supplementary material for: Using Digital Art and Attachment Priming in a Web-Based Serious Game to Reduce Pain and Social Disconnection in Individuals With Chronic Pain and Loneliness: Randomized Controlled Trial
Source: JMIR Serious Games. 2024 Nov 27;12:e52294. doi: 10.2196/52294 (PMC11612526; doi:10.2196/52294)
Supplement: Multimedia Appendix 3 [file games-v12-e52294-s003.docx]

**Table S1.** Participants’ demographic information.

|  | Art-Secure Attachment Condition | Art-Avoidant Attachment Condition | No Art-Secure Attachment Condition | No Art-Avoidant Attachment Condition | Control Condition |
| --- | --- | --- | --- | --- | --- |
| Mean age, y (± SD) | 42.10 ± 11.73 | 42.93 ± 13.84 | 41.25 ± 12.78 | 43.9 ± 13.25 | 43.21 ± 13.80 |
| Female, n (%) | 28 (53.8) | 33 (60.0) | 38 (63.3) | 34 (61.8) | 53 (61.6) |
| Male, n (%) | 20 (38.5) | 19 (34.5) | 22 (36.6) | 19 (34.5) | 32 (37.2) |
| Non-binary, n (%) | 3 (5.8) | 3 (5.5) | 0 (0) | 2 (3.6) | 1 (1.2) |
| Self-described gender, n (%) | 1 (1.9) | 0 (0) | 0 (0) | 0 (0) | 0 (0) |
| American Indian or Alaska Native, n (%) | 1 (1.9) | 1 (1.8) | 2 (3.3) | 0 (0) | 2 (2.3) |
| Asian, n (%) | 4 (7.7) | 3 (5.5) | 4 (6.7) | 3 (5.5) | 3 (3.5) |
| Black, n (%) | 10 (19.2) | 12 (21.8) | 12 (20.0) | 11 (20.0) | 21 (24.4) |
| White, n (%) | 35 (67.3) | 35 (63.6) | 38 (63.3) | 35 (63.6) | 57 (66.3) |
| Multiracial, n (%) | 2 (3.8) | 2 (3.6) | 3 (5.0) | 4 (7.3) | 2 (2.3) |
| Unknown race, n (%) | 0 (0) | 2 (3.6) | 1 (1.7) | 0 (0) | 0 (0) |
| Decline race, n (%) | 0 (0) | 0 (0) | 0 (0) | 2 (3.6) | 1 (1.2) |
| Ethnically Hispanic, any race, n (%) | 3 (5.8) | 2 (3.6) | 6 (10.0) | 3 (5.5) | 6 (7.0) |
| Doctorate degree, n (%) | 2 (3.8) | 1 (1.8) | 1 (1.7) | 2 (3.6) | 2 (2.3) |
| Professional degree, n (%) | 0 (0) | 0 (0) | 0 (0) | 0 (0) | 2 (2.3) |
| Master’s degree, n (%) | 15 (28.8) | 14 (25.4) | 8 (13.3) | 8 (14.5) | 13 (15.1) |
| Bachelor’s degree, n (%) | 14 (26.9) | 17 (30.9) | 24 (40.0) | 22 (40.0) | 27 (31.4) |
| Associate’s degree, n (%) | 7 (13.5) | 1 (1.8) | 12 (20.0) | 6 (10.9) | 12 (14.0) |
| Some college, no degree, n (%) | 5 (9.6) | 10 (18.2) | 6 (10.0) | 9 (16.4) | 18 (20.9) |
| Occupational, technical, or vocational program, n (%) | 2 (3.8) | 7 (12.7) | 2 (3.3) | 5 (9.1) | 4 (4.7) |
| High school graduate or GED, n (%) | 6 (11.5) | 4 (7.3) | 6 (10.0) | 3 (5.5) | 8 (9.3) |
| No high school degree, n (%) | 1 (1.9) | 1 (1.8) | 1 (1.7) | 0 (0) | 0 (0) |
| Working now, n (%) | 30 (57.7) | 28 (50.9) | 31 (51.7) | 28 (50.9) | 45 (52.3) |
| Looking for work, n (%) | 0 (0) | 3 (5.5) | 3 (5.0) | 4 (7.3) | 5 (5.8) |
| Sick leave or maternity leave, n (%) | 2 (3.8) | 0 (0) | 0 (0) | 0 (0) | 1 (1.2) |
| Disabled due to pain, permanently or temporarily, n (%) | 9 (17.3) | 8 (14.5) | 9 (15.0) | 12 (21.8) | 14 (16.3) |
| Disabled for reasons other than back pain, n (%) | 3 (5.8) | 4 (7.3) | 8 (13.3) | 0 (0) | 4 (4.7) |
| Student, n (%) | 1 (1.9) | 3 (5.5) | 1 (1.7) | 0 (0) | 5 (5.8) |
| Temporarily laid off, n (%) | 0 (0) | 0 (0) | 0 (0) | 3 (5.5) | 1 (1.2) |
| Retired, n (%) | 2 (3.8) | 4 (7.3) | 4 (6.7) | 5 (9.1) | 8 (9.3) |
| Keeping the house, n (%) | 1 (1.9) | 3 (5.5) | 3 (5.0) | 1 (1.8) | 3 (3.5) |
| Other work, specify, n (%) | 4 (7.7) | 2 (3.6) | 1 (1.7) | 2 (3.6) | 0 (0) |
| Unknown work, n (%) | 0 (0) | 0 (0) | 0 (0) | 0 (0) | 0 (0) |
| Excellent health, n (%) | 2 (3.8) | 1 (1.8) | 0 (0) | 0 (0) | 1 (1.7) |
| Very good health, n (%) | 9 (17.3) | 8 (14.5) | 18 (30.0) | 8 (14.5) | 12 (20.0) |
| Good health, n (%) | 21 (40.3) | 23 (41.8) | 19 (31.7) | 23 (41.8) | 37 (61.7) |
| Fair health, n (%) | 15 (28.8) | 15 (27.3) | 15 (25.0) | 19 (34.5) | 31 (51.7) |
| Poor health, n (%) | 5 (9.6) | 8 (14.5) | 8 (13.3) | 5 (9.1) | 5 (8.3) |
